# The probabilistic dependence of ship-induced waves is preserved spatially and temporally in the Savannah River (USA)

**DOI:** 10.1038/s41598-024-78924-z

**Published:** 2024-11-15

**Authors:** Patricia Mares-Nasarre, Alexandra Muscalus, Kevin Haas, Oswaldo Morales-Nápoles

**Affiliations:** 1 Delft University of Technology, Hydraulic Structures and Flood Risk, 2628 Delft, CN The Netherlands; 2 Applied Ocean Physics and Engineering, Woods Hole Oceanographic Institution, Woods Hole, MA 02543 USA; 3 School of Civil and Environmental Engineering, Georgia Institute of Technology, Atlanta, GA 30332 USA

**Keywords:** Civil engineering, Applied mathematics

## Abstract

The rapid changes in the shipping fleet during the last decades has increased the ship-induced loads and, thus, their impact on infrastructures, margin protections and ecosystems. Primary waves have been pointed out as the cause of those impacts, with heights that can exceed 2 m and periods around 2 minutes. Consequently, extensive literature can be found on their estimation mainly from a deterministic perspective with methods based on datasets limited to one location, making difficult their generalization. These studies propose either computationally expensive numerical models or empirical equations which often underestimate the extreme primary waves, hindering their use for design purposes. Moreover, a framework to allow the design of infrastructure under ship-wave attack based on probabilistic concepts such as return periods is still missing. In this study, a probabilistic model based on bivariate copulas is proposed to model the joint distribution of the primary wave height, the peak of the total energy flux, the ship length, the ship width, the relative velocity of the ship and the blockage factor. This model, a vine-copula, is developed and validated for four different deployments along the Savannah river (USA), with different locations and times. To do so, the model is quantified using part of the data in one deployment and validated using the rest of the data from this deployment and data of the other three. The vine-copula is validated from both a predictive performance point of view and with respect to the statistical properties. We prove that the probabilistic dependence of the data is preserved spatially and temporally in the Savannah river.

## Introduction

The rapid evolution of the shipping fleet over the past few decades has given rise to concerns about increased ship-induced loads on coastal ecosystems and infrastructure. Between 1900 and 2016, the average capacity of container ships almost quadrupled, and the average capacity of newly built container ships increased by a factor of six^1^. The passage of large ships through water generates a complex wave field, including water level variations caused by the flow field around the ship that are called primary waves. When large ships navigate confined waters, i.e. shipping channels, they can produce long period (typically 2 minutes) primary waves with heights that can exceed 2 m^2–4^. Primary waves can carry massive amounts of energy, threatening the safety of boaters and channel shorelines. Consequently, extensive literature can be found investigating links between these ship-induced primary waves and the increase of the suspended sediment concentration^3,5,6^ and turbidity^7,8^, erosion in waterways^4,9^, and damage to riverine structures^10,11^ and coastal species and natural ecosystems^12,13^, among others. For an overview of the impacts of ship-induced waves, the reader is referred to Ref.^14^.

Given the relevance of the impacts of primary waves, different approaches for estimating primary wave severity are found in the literature, from numerical models to empirical equations. Numerical methods based on Boussinesq^15,16^, shallow-water^2,17^ and incompressible Navier-Stokes equations^18,19^ provide reasonable results for specific cases of study but at a high computational cost. Since these models are highly non-linear, and the local wave characteristics highly depend on the bathymetry, high resolution on both the input data and the numerical mesh together with a large model domain is required. All these factors hinder the use of these numerical models in practical design applications.

Regarding empirical equations^20–28^, these models aim to provide estimations of the depression produced by the passing ship based on the ship and waterway characteristics. They are derived from scale laboratory experiments or field measurements, being limited to the ranges of study analyzed by the authors. Moreover, most of them were developed for artificial inland channels with constant rectangular or trapezoidal cross-sections and water depths. For an overview of the datasets used to derive the empirical equations, the reader is referred to Ref.^28^. Different studies^28,29^ point out the lack of generalization of these empirical models. In Ref.^28^, the equations proposed in^20–27^ were assessed using field measurements in the Stockholm Archipelago (Sweden), obtaining poor results. Later, Ref.^29^, used field data from Savannah river (USA) to assess the performance of these wake formulas. The authors concluded that although simple depression prediction models have a reasonable performance for most of the ship-induced waves, the largest recorded events were consistently underpredicted. Therefore, the extreme events, which are typically used for design, are not properly characterized by the existing empirical equations.

Probabilistic models are widely used to model real-world phenomena^30,31^ due to their advantages in incorporating their uncertainty and natural randomness. Specifically within the Civil Engineering field, probabilistic models based on bivariate copulas have been successfully applied to model the joint probability distribution of sea wave variables^32–34^ or to assess the coastal risk of flooding by modelling the dependence between the different components of water level^35,36^. Also, Ref.^37^, suggested quantifying the multivariate joint distribution of primary wave heights using a Gaussian copula-based model. However, the proposed model could not be generalized and was limited to investigations of the primary waves at a groin tip in the Elbe estuary (Germany). Moreover, the model was limited by the assumption of the Gaussian copula family, which is recognized not to fully describe the dependence of some natural variables^32,38,39^. Therefore, the motivation of the present study is to give a first step toward a generalized probabilistic model for describing the joint multivariate distribution of ship-induced waves. To do so, a copula-based model, a vine-copula, without the preassumption of any copula family is developed. The proposed model can be generalized spatially and temporally in the Savannah river (USA) based on the results of four sets of field observations^29^. Thus, the contribution of this paper is two-fold: (1) develop a multivariate dependence model to describe the uncertainty of ship-induced primary waves using a more flexible model, a vine-copula, and (2) prove that the developed model can be extended to different locations and times within the Savannah river (USA). The resulting model allows the computation of conditional distributions of the ship-induced loads given the characteristics of the passing ship and shipping channel.

The paper is presented in four main sections. First, in the “Methods” section, the field campaign in the Savannah river is described and the procedure to define and validate the probabilistic model is explained. Second, in the “Results” section, the developed model is presented and validated. After that, in the “Discussion” section, the assumptions and limitations of the proposed model are discussed and an example of application is briefly presented. Finally, in the “Conclusions” section, the main conclusions of this study are highlighted.

## Methods

**Fig. 1:**
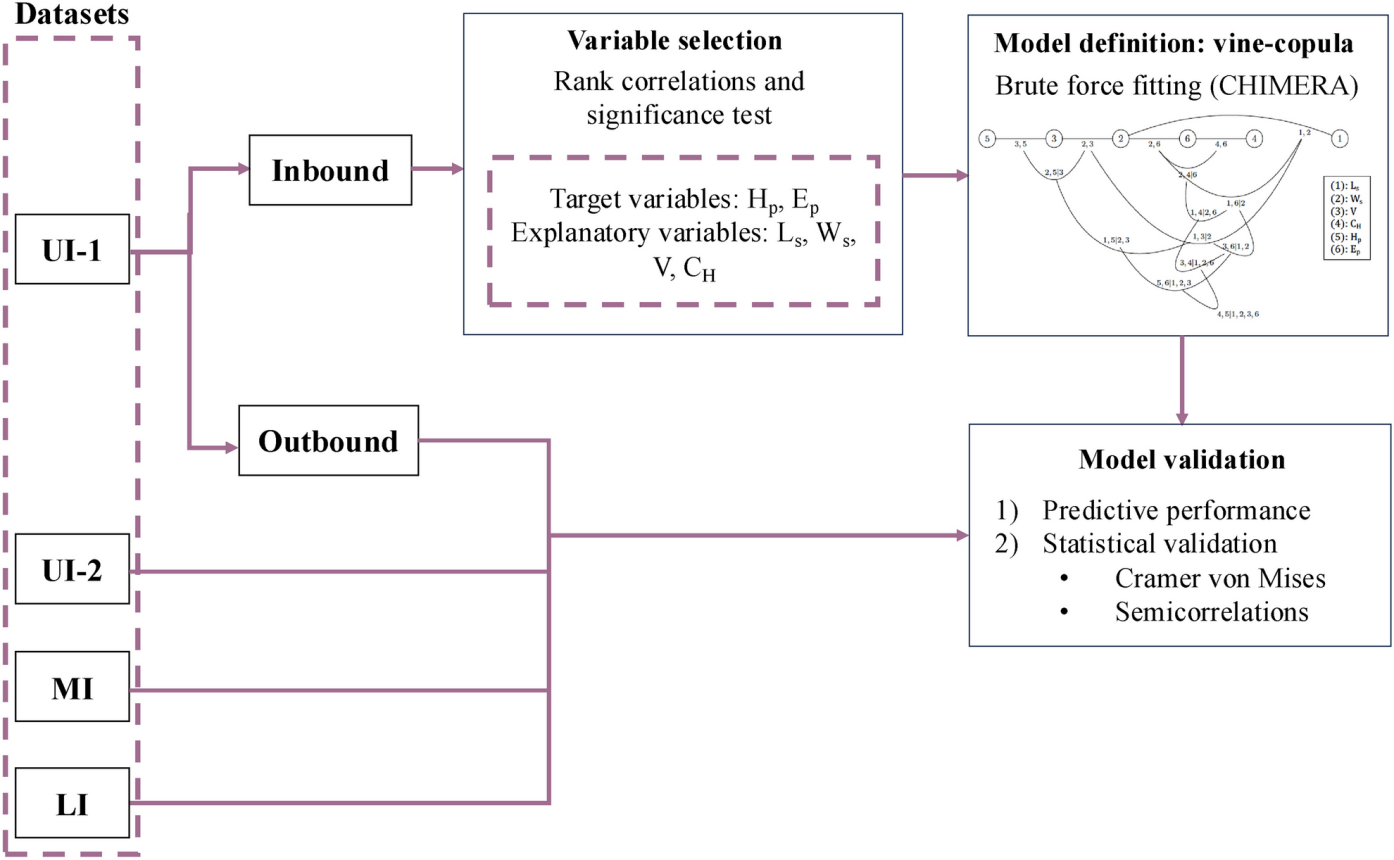
Workflow diagram of the main steps of the current methodology.

Ship-induced waves in the Savannah river were characterized using the four field datasets described in Ref.^29^. These datasets include not only hydrodynamic variables of the ship-wave event (e.g.: primary wave height, $H_p$, and cross-shore ship-wave velocity) but also the characteristics of the ship passage (e.g.: ship dimensions and relative ship velocity). A first dataset was divided into training and testing subsets based on the ship travelling direction (here on, inbound and outbound). The inbound data were used to assess the rank correlations^40^ between the variables in the dataset and determine the best explanatory variables to describe a ship-wave event. Next, a vine-copula^41^ was developed to model the dependence between the selected variables for this training dataset. The obtained model is validated using the testing subset (outbound) and the datasets from the other three field deployments. Figure 1 presents a workflow diagram with the main steps of the methodology, which are explained further in the following sections.

### Field datasets

Primary waves were measured at the margins of the shipping channel located in the Savannah River, a major tidal river with natural, irregular shorelines in the southeastern United States. In the vicinity of the measurements, Bird/Long Island divides the river into the Main (northeastern side) and South (southwestern side) channels. About 25–35% of the Main Channel width is spanned by a shipping channel that provides access to the Port of Savannah for a variety of vessels, including bulk carriers, vehicle carriers, chemical tankers, and container ships. To improve accessibility for deep-draft ships, the shipping channel was deepened from 12.80 to 14.33 m between 2021 and 2022, but repeated bathymetric surveys indicate that the channel margins near measurement locations were largely unchanged by the deepening.

**Fig. 2:**
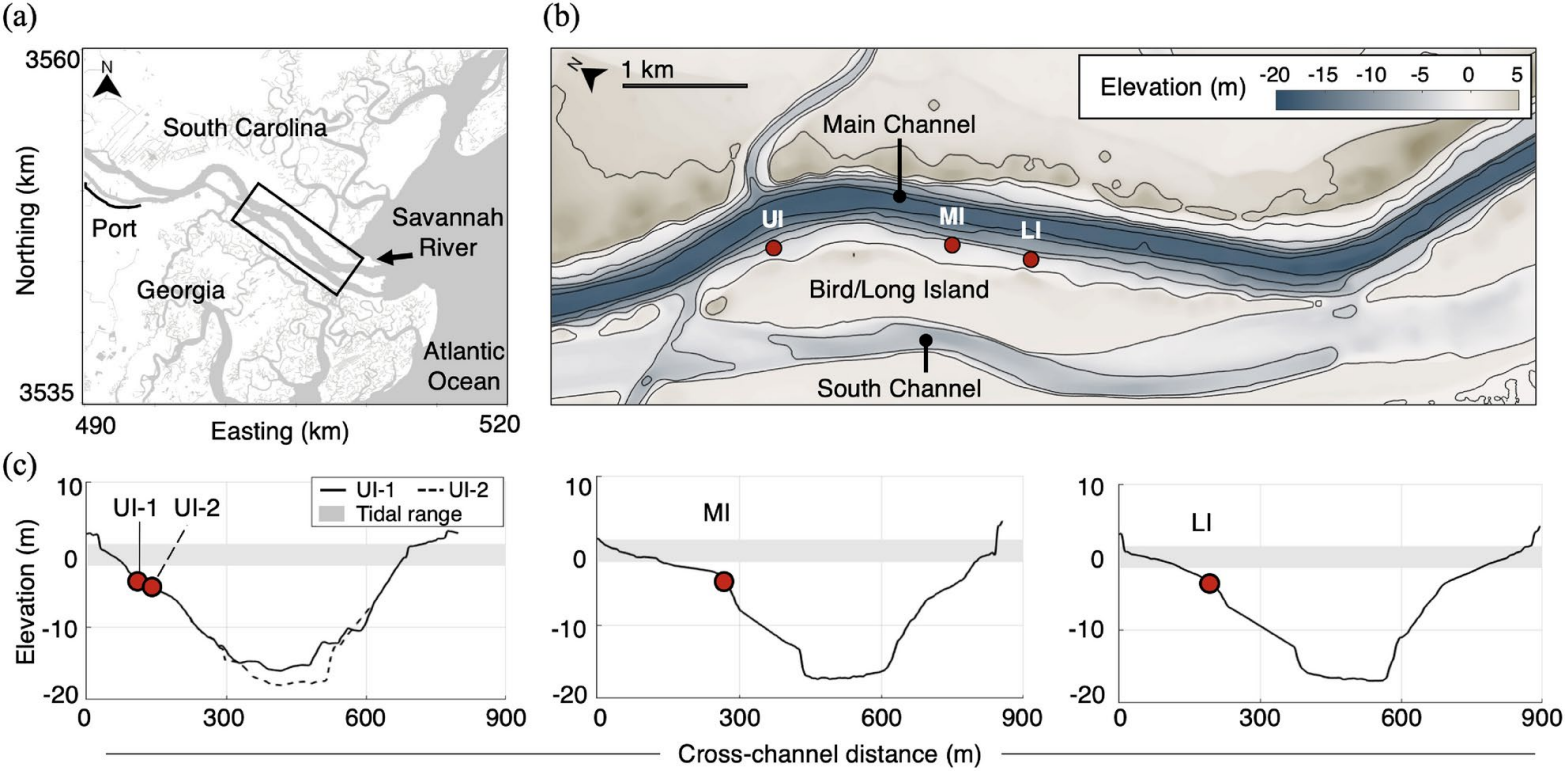
(**a**) General location of the field campaign^29^, (**b**) 2022 topography and bathymetry around the deployment sites (red filled circles) with contours (black line) at 5 m intervals from 5 to $-20$ m, and (**c**) depth profiles of the Savannah River in the locations of the deployments. The boxed region in (**a**) indicates the spatial extents shown in (**b**).

Hydrodynamic observations of primary waves were collected at means depths of 3.5–4.4 m with pressure transducers and/or Nortek Aquadopp velocimeters through four deployments (Fig. 2): Upper-Island 1 (UI-1) with 179 recorded events, Upper Island 2 (UI-2) with 47 recorded events, Mid-Island (MI) with 34 recorded events, and Lower-Island (LI) with 36 recorded events. The channel depth profiles displayed in Fig. 2c show similar cross-sectional areas at the four sites. However, cross-sectional mean depths vary among sites by up to 2.2 m (about 25%), and bathymetric features differ due to the natural variability of the river. For instance, there are broad, shallow shelves along Bird/Long Island at the locations of MI and LI, but not at UI. In addition, whereas UI-1 observations were obtained in 2017, prior to channel deepening, all other observations took place in 2022 (from February to June), post-deepening.

Each dataset includes 1 Hz water surface elevations, from which wave heights and tidal stages are computed. The UI-1 and LI data also contain 1 Hz fluid velocities, from which wave energy flux is directly computed. All hydrodynamic data were band-pass filtered to separate primary wave signals from wind waves, Kelvin wake, and tidal processes. Ship properties and tracks were obtained from Automatic Identification System (AIS) data accessed via multiple sources: Marine Cadastre AccessAIS, ShipTracks.com, FleetMon.com, and US Army Corps of Engineers. Details of hydrodynamic and AIS data processing are provided by^29^.

Using the AIS ship tracks to identify passage times, individual primary wave events were quantified with an automatic detection algorithm and manually verified. The primary wave was defined with water level zero down-crossings, beginning just before the depression and ending just after the surge.

### Descriptors of the ship-wave event

Based on the existing literature, two target variables to represent ship-wave events were selected: primary wave height ($H_p$, cm) and the peak of the total energy flux ($E_p$, kW/m) of the ship-wave event. As previously mentioned, the UI-1 dataset is divided into training and testing subsets based on the ship travelling direction (UI-1 inbound and UI-1 outbound, respectively). The Spearman’s rank correlation coefficient^40^ is calculated in pairs between these two variables and each variable in the UI-1 inbound dataset. Rank correlation coefficient assesses the strength of monotonic dependence between two random variables and was used here to identify the best explanatory variables for $H_p$ and $E_p$ within the available variables. Moreover, *p*-values associated with those rank correlations were computed to identify whether the computed correlations were significant. A summary of those results is presented in Fig. 1 in Supplementary Material. Based on the aforementioned analysis, four explanatory variables were identified and included in the model together with $H_p$ and $E_p$. The six total variables included in the model are:

- Primary wave height ($H_p$, cm), defined as the vertical distance between the minimum and maximum point of the primary wave: most empirical formulas are focused on predicting the primary wave drawdown^20–28^, which is defined as the vertical distance between the mean water level and the minimum point of the oscillation. However, the complete height of the primary wave events is better correlated to the energy of the event^29^ and is more relevant for assessing the erosion of the channel margins^4^ or the damage on groynes due to overflow^11,37^.
- Peak of the total energy flux ($E_p$, kW/m), defined as the peak total magnitude of cross-shore and long-shore energy flux per meter of shoreline: hydrodynamic power has been previously used as a proxy for erosion^4^, and is thus a relevant variable to describe the ship-induced load.
- Ship length ($L_s$, m): this variable is included in most empirical equations for assessing the ship-induced drawdown^21,23,25^. Moreover, larger ships have been associated with larger primary ship waves in various studies^28,29,42^.
- Ship width ($W_s$, m): different studies have discussed the influence of ship dimensions, including variables related to the ship width such as the ship cross-section^20–22,24–27^. It should be also noted that ship length and ship width are more accessible and reliably accurate in AIS data than ship draft or cross-section.
- Relative velocity of the ship (*V*, m/s), defined as the difference between the velocity over ground of the ship and the estimated tidal current: relative velocity is a widely accepted variable that governs the ship-induced loads^20–28,37^. Moreover, not only the larger but also the faster ships have been found to produce larger primary waves^29,42^. Consequently, ship velocity restrictions have been applied in some waterways to limit the impact of ship-induced loads^10^.
- Blockage factor ($C_{H} = D_{s} \cdot W_{s} /A_{{channel}}$ (-), where $D_s$ is the ship draft and $A_{channel}$ is the cross-sectional area of the waterway). This variable accounts for the portion of water volume displaced by the passing ship. Thus, it influences the generated ship wave magnitude and is commonly considered in primary wave observational and experimental studies^23,37^.

Table 1 provides an overview of the statistics of the selected variables defined above, as well as the minimum passing distance between the ship and the shore.

**Table 1:** Summary of the statistics of the main variables in the datasets used in this study. The minimum value (min), the mean value (mean), the maximum value (max) and the coefficient of variation (CV) are provided.

Dataset	Primary wave height (cm)				Peak of the total energy flux (kW/m)				Ship length (m)			
	Min	Mean	Max	CV(%)	Min	Mean	Max	CV(%)	Min	Mean	Max	CV(%)
UI-1	1	65	181	59%	0.1	10.2	56.5	93%	106	265	367	24%
UI-2	5	32	87	71%	–	–	–	–	134	259	366	32%
MI	9	32	90	63%	–	–	–	–	172	252	366	27%
LI	5	24	72	69%	0.4	4.0	29.5	138%	123	237	366	30%

Dataset	Ship width (m)				Relative ship velocity (m/s)				Blockage factor (-)			
	Min	Mean	Max	CV(%)	Min	Mean	Max	CV(%)	Min	Mean	Max	CV(%)
UI-1	17	36	48	20%	3.6	6.2	7.9	11%	0.016	0.061	0.103	32%
UI-2	23	36	52	23%	3.9	5.9	8.2	15%	0.024	0.057	0.101	40%
MI	25	35	51	22%	4.6	6.3	7.5	12%	0.024	0.053	0.096	36%
LI	21	35	49	25%	4.4	6.0	7.0	11%	0.023	0.058	0.103	39%

Dataset	Minimum distance ship to shore (m)											
	Min	Mean	Max	CV(%)								
UI-1	287	342	426	7%								
UI-2	304	349	458	8%								
MI	406	496	555	8%								
LI	356	441	526	9%								

### Dependence model definition and validation

A vine-copula^41^ is used to model the probabilistic dependence between the six selected variables, namely $L_s$, $W_s$, *V*, $C_H$, $H_p$ and $E_p$. A vine-copula model is composed of a series of nested trees which model the dependence in bivariate pieces. In the first tree, each node represents one variable, and each arc represents the dependence between the two variables and is quantified using a bivariate copula. In the subsequent trees, the nodes are the bivariate copulas in the previous tree, and the arcs are quantified with conditional bivariate copulas that connect nodes with a common variable. Thus, the definition of a vine-copula involves defining both the graph, named regular vine, and the fitted copulas that model the dependence between each (un)conditional pair. For six variables, 23,040 regular vines (graphs) are possible^43^. Here, all the possible regular vines are fitted using the atlas Chimera^44^, and the best model in terms of Akaike Information Criterion^45^ (*AIC*) is selected. All the bivariate copula families included in Ref.^46^ are considered.

Once the vine-copula is selected and fitted using the UI-1 inbound dataset, it is validated using datasets collected in different field campaigns from two perspectives: (1) predictive performance, and (2) statistical properties validation. From the predictive performance perspective, the joint probabilities observed in each dataset are compared to those predicted by the model. In the case of the UI-2 and MI datasets where the variable $E_p$ is not available, the adopted dependence model is the same as that obtained for UI-1 inbound but removing $E_p$, named here the “reduced” model. Some variable pairs in the reduced model are not explicitly present in the complete model due to the presence of $E_p$ in the second. In these scenarios, the copula model is obtained from the same unconditional terms in the complete model. For example, the pairing between $C_h$ and $W_s$ requires to be defined in the reduced model, but this pair was not present in the complete model due to the presence of $E_p$. Thus, the pair $C_h$ and $W_s$ is quantified in the reduced model using the conditional pair $C_h$ and $W_s$ given $E_p$ from the complete model. The agreement between the observed and predicted non-exceedance joint probabilities is assessed using the coefficient of determination ($R^2$). The value of $R^2$ ( $0 \le R^2 \le 1$) indicates (roughly) the portion of variance explained by the model: the higher the value of $R^2$ , the better the agreement.

To perform the statistical validation, a list of regular vine-copula models to compare with the proposed models (both complete and reduced) are defined. First, the best model in terms of *AIC* for each dataset (except UI-1 inbound) is determined using Brute Force, similar to the definition of the complete proposed model. These regular vine-copulas are denoted here as $BF_{dataset \ id}$. Second, the regular vine (graph) from the proposed model is fitted to each dataset (except UI-1 inbound). The obtained regular vine-copulas are denoted here as $MM_{dataset \ id}$. These models are compared with the proposed models in order to determine whether there is a significant difference between the proposed models and the best models that can be obtained for each dataset (here, *BF* and *MM* models). Therefore, if no significant differences can be observed, the proposed dependence models are applicable to every dataset and, thus, the probabilistic dependence is preserved regardless the location and time of the deployment. In order to compare *BF* and *MM* with the proposed models, two goodness of fit techniques are used: Cramer-von-Mises Statistic^47^ ($S_{CvM}$) with its p-value ($p_{CvM}$) and semicorrelations^48^. $S_{CvM}$ assesses the distance between two multivariate distributions; a perfect fit is given by $S_{{CvM}} \to 0$. A hypothesis test is performed with $S_{CvM}$, where the null hypothesis is that both distributions are the same. The value of $p_{CvM}$ is then computed following the procedure described in Ref.^47^, which indicates that $p_{CvM}<0.05$ (significance level) means it can be rejected that both distributions are the same. Semicorrelations are used to evaluate the presence, or lack thereof, asymmetries in each bivariate pair. This approach consists of computing the Pearson’s correlations^49,50^ in four quadrants delimited using $X=Y=0$ after transforming the variables to standard normal space. This procedure is applied to the random samples obtained from the fitted models, and similar semicorrelations indicate that the models predict similar asymmetries. Therefore, $S_{CvM}$ compares the whole multivariate distribution, while semicorrelations analyze the dependence “shape” of each pair.

## Results

In this section, the vine-copula obtained to model the dependence of ship-generated waves in dataset UI-1 inbound is presented. After that, the results of the validation using the missing datasets are described. Finally, although not the main focus of the current study, the univariate marginal distributions of each variable are compared, and parametric models are proposed. The quantification of the marginals is addressed here to propose a complete model that can be used in subsequent research, which allows for the inference of events that have not been observed yet.

### Dependence model for ship-waves

**Fig. 3:**
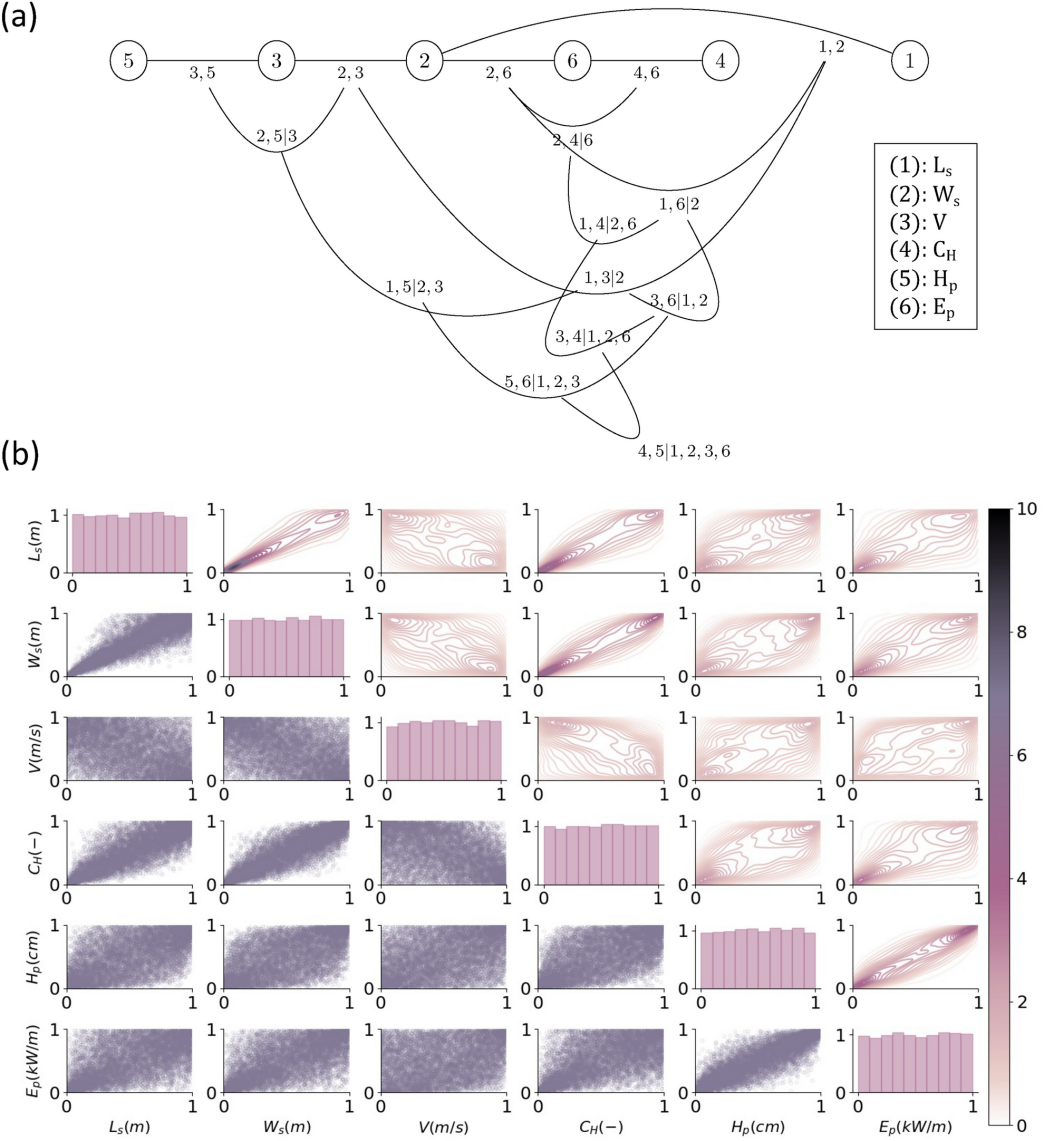
Dependence model: (**a**) regular vine, and (**b**) scatter matrix with 10,000 samples and probability densities.

As previously described, the proposed dependence model is a vine-copula defined using the inbound ships of UI-1 dataset. Fig. 3a presents the graph of the complete model, while Fig. 3b shows the scatter plots and densities of the joint distribution given by the model. In Fig. 2 in Supplementary material, the decomposition of the regular vine-copula in dependence trees can be found, together with the parametric bivariate copulas used to quantify them. Moreover, Fig. 4 in Supplementary material presents the equivalent information for the reduced regular vine-copula.

### Validation of the dependence model

**Fig. 4:**
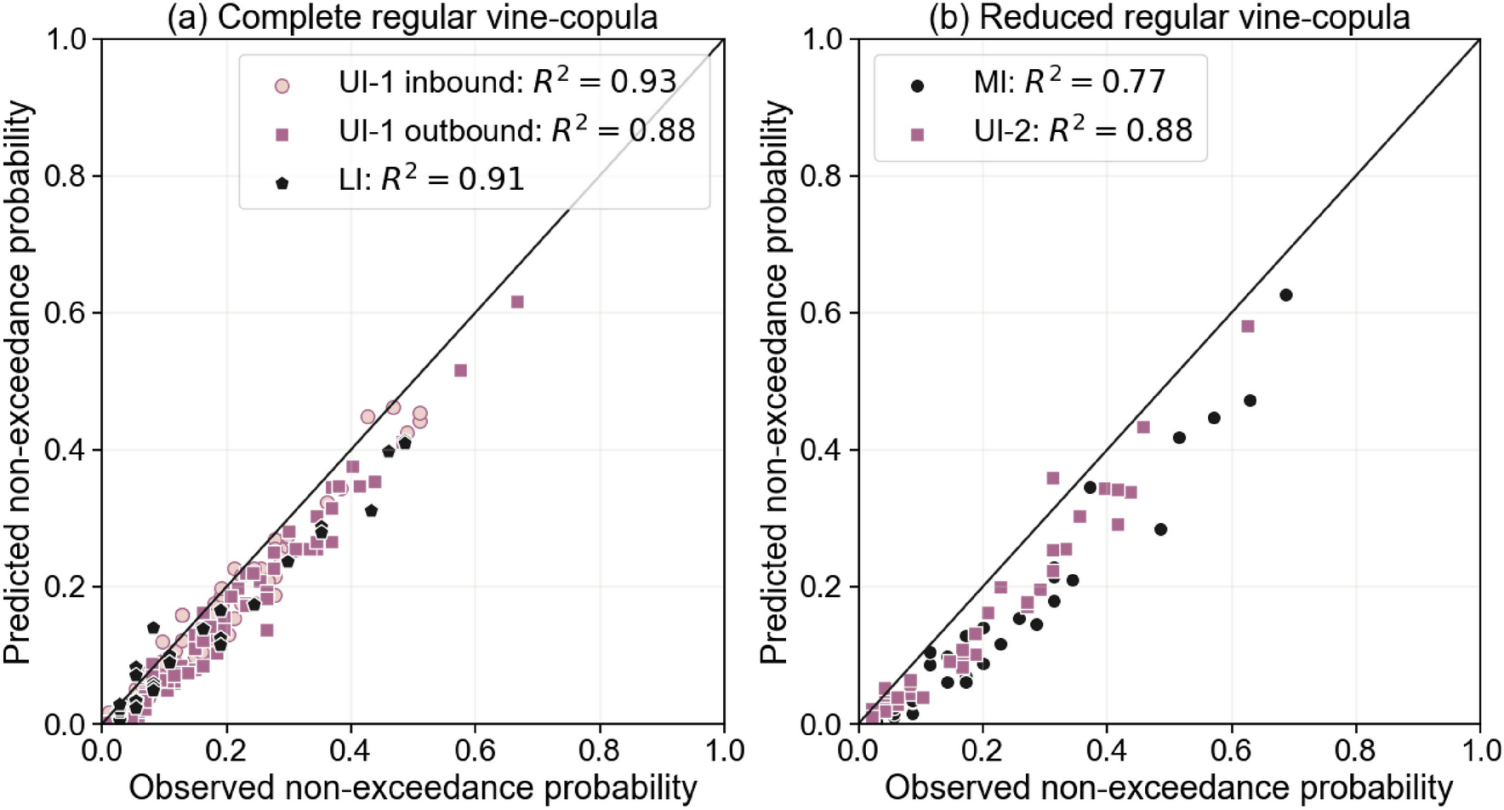
Comparison of the observed and predicted non-exceedance probabilities: (**a**) databases with measurements of $E_p$ against full regular vine-copula ($P\left[ {L_{s} \le x_{1} ,W_{s} \le x_{2} ,V \le x_{3} ,C_{H} \le x_{4} ,H_{p} \le x_{5} ,E_{p} \le x_{6} } \right]$), and (**b**) databases without measurements of $E_p$ against reduced regular vine-copula ($P \left[ {L_{s} \le x_{1} ,W_{s} \le x_{2} ,V \le x_{3} ,C_{H} \le x_{4} ,H_{p} \le x_{5} } \right]$).

As previously described, the obtained dependence model is generalized to different times and locations in Savannah river by validating it on the available databases. With regards to predictive performance, both the complete and reduced models provide satisfactory results compared to studies in the literature^28^ when estimating the observed non-exceedance probabilities in the validation datasets (UI-1 inbound, UI-2, MI, and LI). As shown in Fig. 4, $R^2>0.88$ for the complete model and $R^2>0.77$ for the reduced model are obtained. Besides the agreement, some bias can be observed in Fig. 4; the observed non-exceedance probabilities are higher than the predicted ones, especially for the reduced model. However, it should be noted that no fitting process was applied to define the reduced model.

Regarding the statistical validation, first, the proposed models are compared to the best model in terms of *AIC* for each dataset, denoted here as $BF_{dataset \; id}$. $S_{CvM}$ and $p_{CvM}$ are computed between the complete and reduced vine-copula model and the *BF* models. Using a significance level of $\alpha = 0.05$, it cannot be rejected that the multivariate distributions defined by the *BF* models are coming from the multivariate distribution defined by the proposed model. Semicorrelations are also computed to compare the dependence shape of each bivariate pair, and very small differences were observed for most of the bivariate pairs and datasets. Between 85 and 93% of the elements in each comparison between two datasets presented the same sign, and between 82 and 88% of the elements presented absolute differences below 0.2. The differences in the semicorrelations clustered around different variables and quadrants for the different datasets. With all *BF* models, differences were observed for the pairs with $L_s$ in the upper right quadrant (high values of both variables). For $BF_{outbound}$, some differences were also present in the pairs with $L_s$ in the lower left quadrant (low values of both variables). For $BF_{LI}$ and $BF_{UI2}$, differences clustered in the lower left quadrant for the variables $E_p$ and $H_p$, respectively. Differences in the lower right quadrant (low values of variable mentioned here and high values of the other variables) were also present for the model $BF_{UI2}$ for the pairs with the variables *V* and $H_p$. Finally, for the model $BF_{MI}$, differences clustered mainly around the upper right quadrant. Overall, this indicates that the best fitting model according to *AIC* for each dataset is not significantly different to the proposed models and, thus, the dependence structure can be assumed to be the same.

Second, the vine-copula models fitted to each dataset (except UI-1 inbound) preserving the same regular vine (graph) are compared ($MM_{dataset \ id}$) with the proposed models. $S_{CvM}$ and $p_{CvM}$ are computed between the complete and reduced vine-copula model and the *MM* models. Using a significance level of $\alpha = 0.05$, it cannot be rejected that the multivariate distributions defined by the *MM* models are coming from the multivariate distribution defined by the proposed model. Semicorrelations are also computed, and very small differences were observed for most of the bivariate pairs and datasets. Between 83 and 93% of the elements in each comparison between two datasets presented the same sign, and between 83 and 92% of the elements presented absolute differences below 0.2. The differences in the semicorrelations clustered around different variables for the different databases without demonstrating a common pattern. With $MM_{outbound}$, $MM_{MI}$ and $MM_{UI2}$, differences were mainly on the pairs with $L_s$ in the upper right quadrant (high values of both variables). For $MM_{UI2}$, there were also some differences in the lower left quadrant (low values of both variables) in the variable pairs with $H_s$. With $MM_{LI}$, most of the differences appeared in the pairs with *V* in different quadrants and with $E_p$ in the lower left quadrant. Thus, no significant differences are found between the *MM* models, and the proposed models in this study indicated that, given the regular vine (graph), no significant differences are observed in the fitted copulas used to quantify it.

In conclusion, with all the above, it can be concluded that no significant differences are observed in the multivariate distributions of the different datasets and, thus, it can be assumed that regardless the location and time of the data explored, the probabilistic dependence is preserved in the Savannah river.

### Marginal distributions

**Fig. 5:**
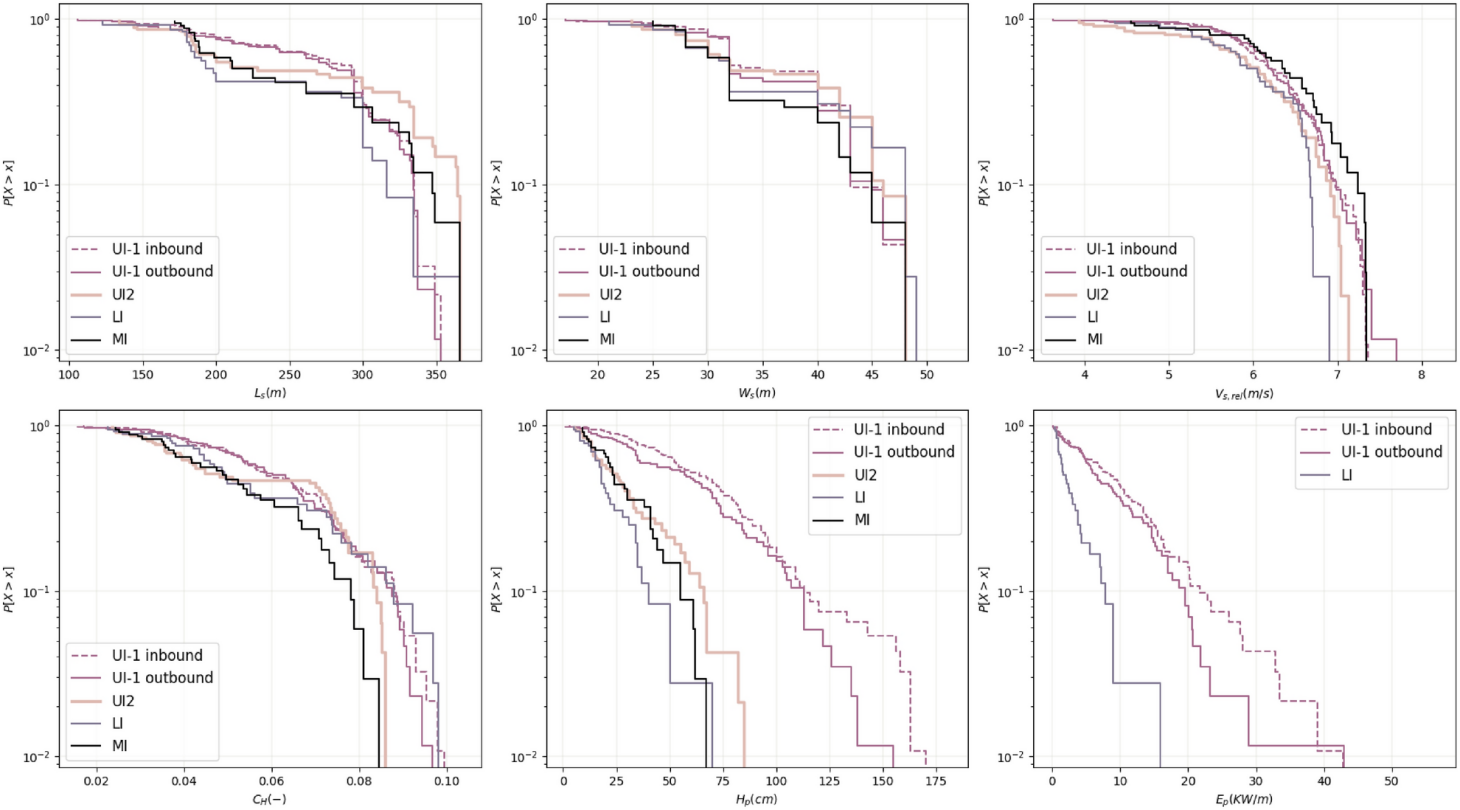
Comparison of the cumulative distribution functions of each studied variables for each dataset. Exceedance plot shown in semi-log space.

In this section, the univariate empirical distribution of each studied variable is modelled using parametric distribution functions. These parametric margins allow for inference of probabilities that have not been observed in the dataset and, thus, perform predictions for the future. An overview of the empirical cumulative distribution functions for each variable and dataset is presented in Fig. 5 as the exceedance plots in semi-log scale.

In order to better assess whether the marginal distributions for the different datasets come from the same distribution function, the two-sided Kolmogorov-Smirnov hypothesis test^51,52^ is applied to compare variable distributions among pairs of datasets. Complete results from the performed tests are available in Supplementary Information in Tables 1, 2, 3, 4, 5 and 6. For the variables $W_s$, *V* and $C_H$, in general, it could not be rejected that the distributions for the different datasets come from the same distribution function. Regarding $L_s$, significant differences are obtained between the UI-1 dataset, both inbound and outbound, and LI dataset. However, visual inspection suggests that all datasets could be modelled using the same parametric distribution function (see top left panel in Fig. 5). With regard to $H_p$, the observations of $H_p$ in the UI-1 dataset cannot be assumed to follow the same distribution as those in UI-2, LI and MI datasets. Thus, two different marginal distributions need to be fitted: one for UI-1 database and one for UI-2, LI and MI databases. Finally, $E_p$ measurements are only available in UI-1 and LI deployments and a statistically difference between them is obtained, according to Kolmogorov-Smirnov test. This result aligns with the findings in^29^; $H_p$ is a good predictor of $E_p$ so similar behavior is expected from both variables. Then, given that the distributions of $H_p$ for UI-1 and LI are statistically different, it is expected that $E_p$ presents the same behavior.

**Fig. 6:**
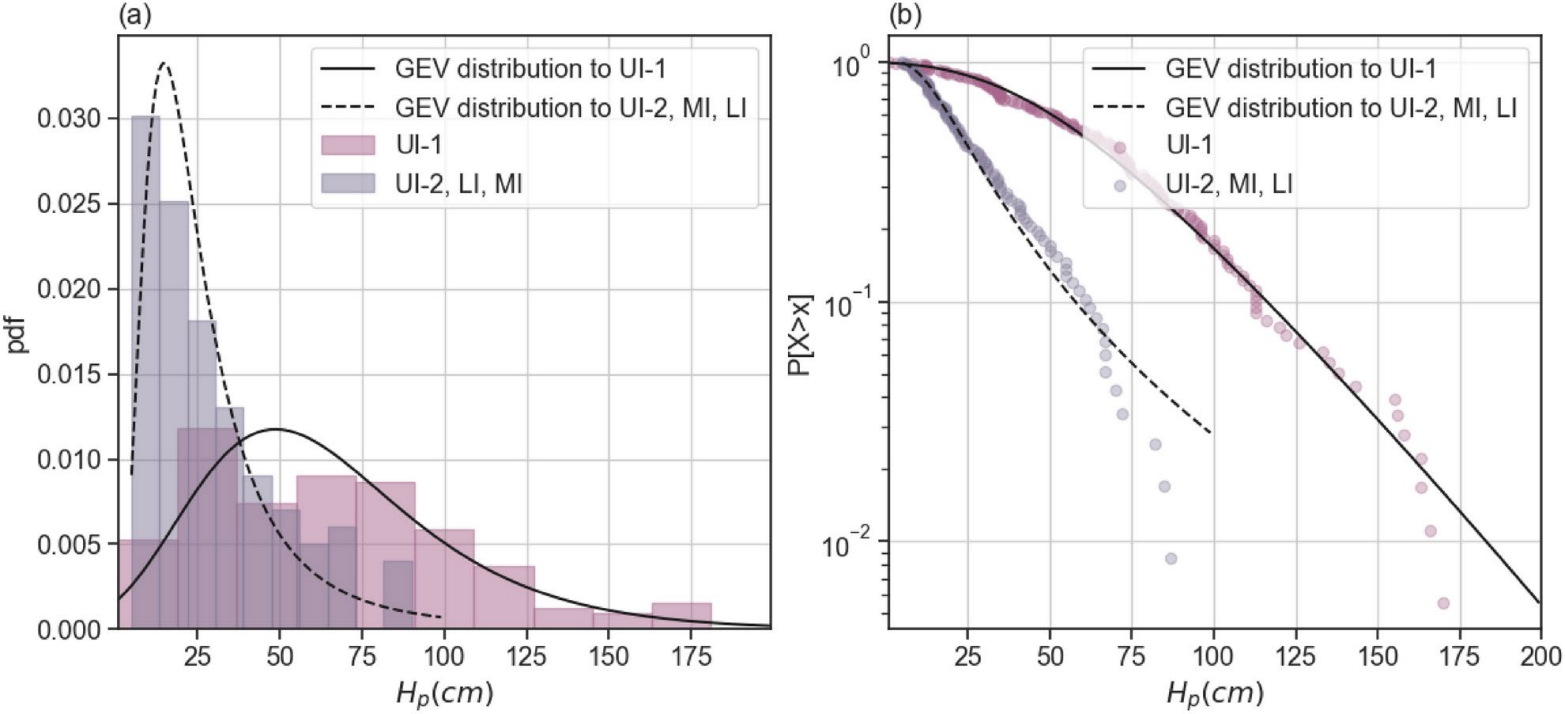
Comparison of the empirical and GEV parametric distribution of $H_{p}$ (cm): (**a**) probability density function, and (**b**) exceedance plot in semi-log scale.

**Fig. 7:**
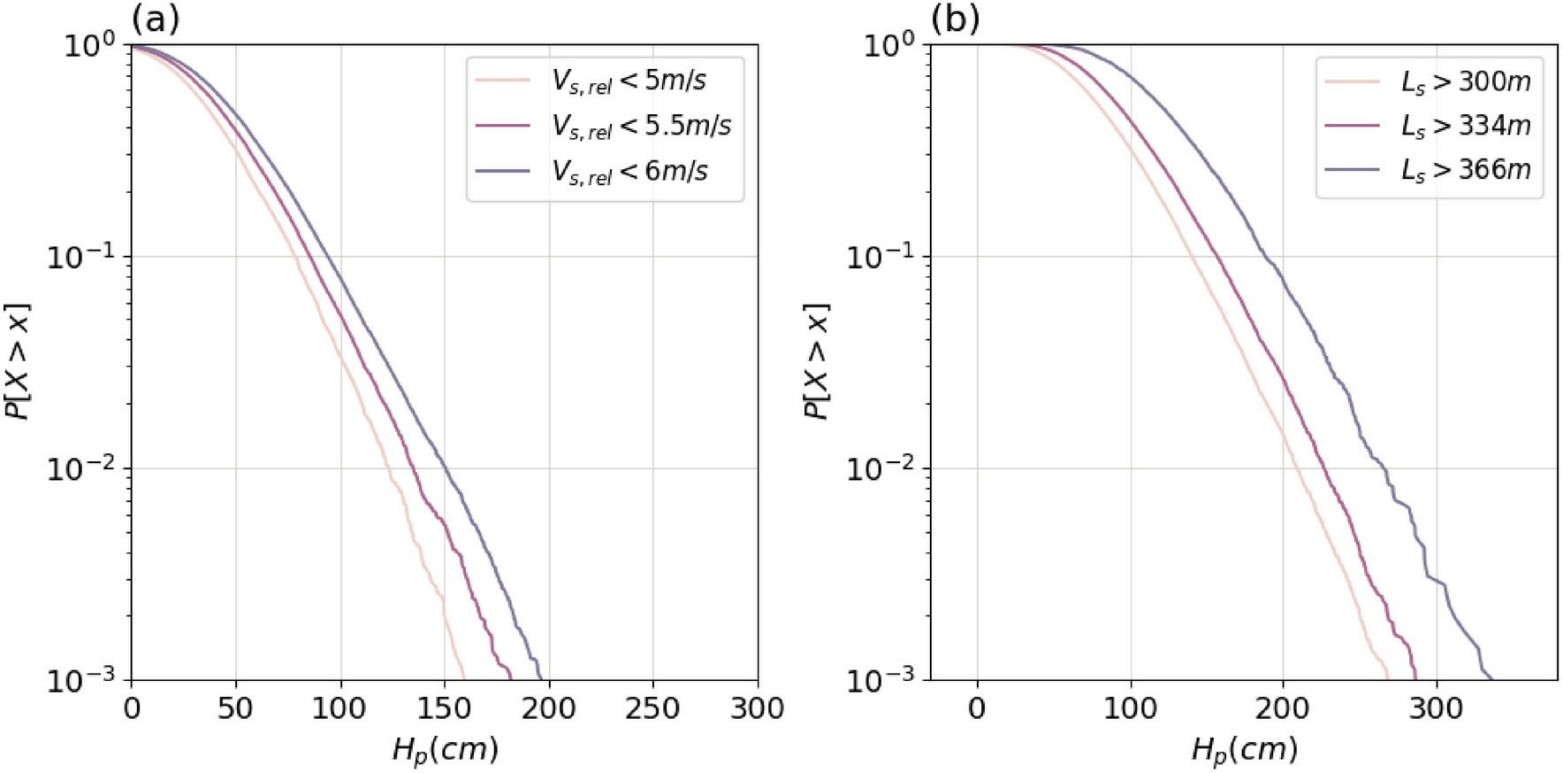
What-if simulated scenarios by the proposed model: (**a**) influence of ship speed limitation on $H_p$, and (**b**) influence of $L_s$ on $H_p$. Marginal distributions at location UI-1 from Table 7 in Supplementary information.

Based on the previous analysis, the variables from different datasets that presented a similar marginal distribution are further analyzed together. Parametric univariate distribution functions are fitted to the observations using Maximum Loglikelihood Estimator, as implemented in Scipy Python package^53^. The following parametric distribution functions are included in the analysis: Lognormal, Normal, Exponential, Generalized Extreme Value (GEV), Gumbel, Generalized Pareto (GPD), Beta, Rayleigh, Uniform, Gamma, Pareto, truncated Normal, and t. All the listed distributions are fitted to the observations. Afterwards, the five best fitting distributions to each dataset based on *AIC* are visually inspected using the exceedance plot in semi-log scale to better visualize the fitting of the distribution to the tail of the distribution. The variables $L_s$, $W_s$, *V* and $H_p$ are modelled using a GEV, $C_H$ is modelled using a Beta distribution and $E_p$ is modelled using a GPD. Figure 6 presents an example of the fitting of GEV distribution to the measurements of $H_p$. The summary of such fitting is given in the Supplementary Information in Table 7, while an illustration of the fit together with the equations for the fitted distributions are provided in Supplementary Information in Figs. 5, 6, 7, 8, and 9.

Figure 6 illustrates the fit of the Generalized Extreme Value (GEV) distribution (cumulative distribution function in Eq. (1) in Supplementary Information) with parameters given in Table 7 to the observations of $H_{p}$.

## Discussion

In the present study, a vine-copula for describing the energy and height of ship-induced primary waves is developed considering only ship dimensions ($L_s$ and $W_s$), *V*, and $C_H$. This model is validated using datasets with different locations and deployment times along the Savannah river, demonstrating that no significant differences are observed between the dependence structure in the datasets. The proposed model, in contrast with previous studies which relied in the measurements in a single location and time^22,25,26,28,37^, takes a step toward the generalisation of the models to describe primary ship waves. Moreover, the proposed model does not require any assumptions or simplification in the ship-fluid interaction or hydrodynamic conditions around it, in contrast to numerical models^15–17^. However, it should be noted that the proposed model can only be generalized to the margins of the Savannah river, and future research should focus on gathering datasets in other locations and conditions to further validate and generalize the model. As pointed out in previous studies^14,29^, there is scarce field data on ship-induced waves, making difficult to define a global model which describes the behavior of this phenomenon.

Regarding the model definition, here two target variables were selected to describe the ship-induced loads: $H_p$ and $E_p$. These variables were selected based on the potential use of the model for erosion assessment^4^ or damage to riverine structures^11^. However, different variables might be more relevant for other applications. In those cases, the same procedure in “Dependence model definition and validation” can be applied to define a new model. Once the target variables were selected, four more explanatory variables ($L_s$, $W_s$, *V*, and $C_H$) were chosen based on the existing literature and the observed rank correlations with the target variables, as explained in section “Descriptors of the ship-wave event”. It should be noted that these variables were selected based on the available observations and, thus, limited to the observed ranges in the field campaign. For instance, several studies in literature^8,28^ point out the relevance of the bathymetry or the ship passage distance (distance between the measurement point and the ship) to describe the primary waves. However, these two variables did not demonstrate significant correlations with the target variables in this study, and thus, they were not included in the model. Hence, further effort should be made to build a broader dataset with different conditions and ranges of the variables.

The proposed model can potentially be used to provide useful information to decision-makers and better assess the influence of the regulations in the waterway. For instance, Fig. 7a presents the influence of speed limitation on the generated $H_p$. As expected, lower velocity limits lead to smaller generated waves. With the current changes in the shipping fleet, the model can also be used together with the projections on the evolution of the ship dimensions to infer the future ship-induced loads that will need to be faced. For instance, Fig. 7b depicts the influence of large ships (large $L_s$) on the generated $H_p$. Moreover, if the traffic composition is modified, the proposed dependence model can still be used by changing the marginal distributions of the ship dimensions.

## Conclusions

The probabilistic dependence between $E_p$, $H_p$, $L_s$, $W_s$, *V*, and $C_H$ is preserved in the different locations and deployment times included in this study along the Savannah river. Thus, a single probabilistic model to describe such dependence is proposed. This model can be used to support the development and assessment of waterway regulations in the Savannah river. Field data on ship-induced waves is scarce, hindering the development of a global model which describes the behavior of this phenomenon. Thus, future research should focus on gathering datasets in other locations and conditions (e.g.: bathymetry and channel geometry) to further validate and generalize the model.

## Supplementary Information

Supplementary Information.

## Data Availability

Data will be provided under reasonable request to the corresponding author (Patricia Mares-Nasarre).
