# Supplementary material for: The probabilistic dependence of ship-induced waves is preserved spatially and temporally in the Savannah River (USA)
Source: Sci Rep. 2024 Nov 15;14:28154. doi: 10.1038/s41598-024-78924-z (PMC11567961; doi:10.1038/s41598-024-78924-z)
Supplement: Supplementary file 1 — Supplementary Information. [file 41598_2024_78924_MOESM1_ESM.pdf]

Supplement to: The probabilistic dependence of ship-induced waves is preserved spatially and temporally in the Savannah River (USA)

Patricia Mares-Nasarre[1,*], Alexandra Muscalus[2], Kevin Haas[3], and Oswaldo Morales-Nápoles[1]

[1]Delft University of Technology, Hydraulic Structures and Flood Risk, Delft, 2628 CN, the Netherlands
[2]Applied Ocean Physics and Engineering, Woods Hole Oceanographic Institution, Woods Hole, MA 02543, USA.
[3]School of Civil and Environmental Engineering, Georgia Institute of Technology, Atlanta, GA 30332, USA.
[*]p.maresnasarre@tudelft.nl

ABSTRACT

The rapid changes in the shipping fleet during the last decades has increased the ship-induced loads and, thus, their impact on infrastructures, margin protections and ecosystems. Primary waves have been pointed out as the cause of those impacts, with heights that can exceed 2m and periods around 2 minutes. Consequently, extensive literature can be found on their estimation mainly from a deterministic perspective with methods based on datasets limited to one location, making difficult their generalization. These studies propose either computationally expensive numerical models or empirical equations which often underestimate the extreme primary waves, hindering their use for design purposes. Moreover, a framework to allow the design of infrastructure under ship-wave attack based on probabilistic concepts such as return periods is still missing. In this study, a probabilistic model based on bivariate copulas is proposed to model the joint distribution of the primary wave height, the peak of the total energy flux, the ship length, the ship width, the relative velocity of the ship and the blockage factor. This model, a vine-copula, is developed and validated for four different deployments along the Savannah river (USA), with different locations and times. To do so, the model is quantified using part of the data in one deployment and validated using the rest of the data from this deployment and data of the other three. The regular vine-copula is validated from both a predictive performance point of view and with respect to the statistical properties. We prove that the probabilistic dependence of the data are preserved spatially and temporally in the Savannah river.

Rank correlation matrix for UI-1 inbound dataset

As part of the analysis to determine the explanatory variables for the primary wave height and the total peak of energy flux, the Spearman's rank correlation coefficient[?] is computed between each pair of variables in the UI-1 inbound dataset. Moreover, p-values associated with those rank correlations were computed to identify whether the computed correlations were significant.

Dependence models

Complete regular vine-copula model

Here, the tree decomposition of the regular vine-copula for UI-1 inbound is presented in Fig. 2.

The quantification of each dependence tree in the complete regular vine-copula model with bivariate copulas is given by:

Tree: 1
4,6 <-> Joe 180°, parameters = 2.23529
5,3 <-> Gaussian, parameters = 0.377419
3,2 <-> Frank, parameters = -2.41646
1,2 <-> BB7 180°, parameters = [5.70257, 1e-06]
2,6 <-> Gumbel 180°, parameters = 1.77111
Tree: 2
4,2 | 6 <-> Gaussian, parameters = 0.8069
5,2 | 3 <-> Gaussian, parameters = 0.828941
3,1 | 2 <-> Independence
1,6 | 2 <-> Independence
Tree: 3

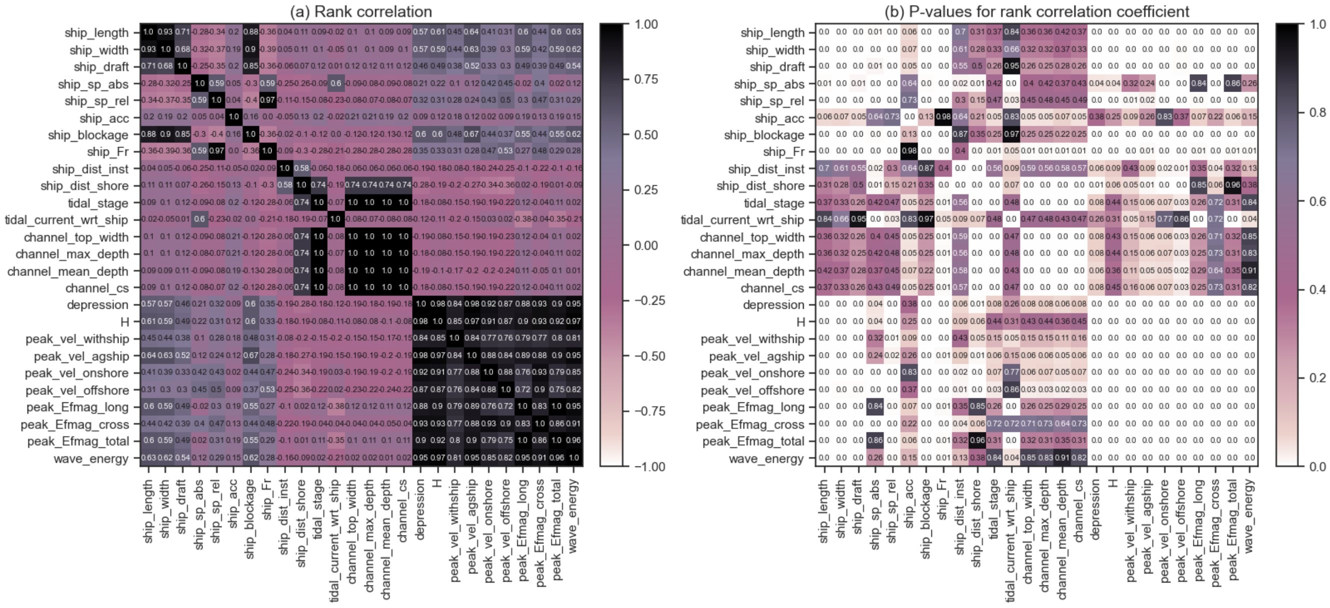

Figure 1. Rank correlation matrix for UI-1 inbound dataset: (a) Spearman's rank correlation coefficient, and (b) p-values. Note that p-values < 0.05 indicate a significant dependence.

4,1 | 2,6 <-> BB1 180°, parameters = [1e-06, 1.20484]
5,1 | 2,3 <-> BB1 180°, parameters = [1e-06, 1.23315]
3,6 | 1,2 <-> Gaussian, parameters = 0.765358
Tree: 4
4,3 | 1,2,6 <-> Gaussian, parameters = -0.265501
5,6 | 1,2,3 <-> Frank, parameters = 3.96752
Tree: 5
4,5 | 3,1,2,6 <-> Clayton 180°, parameters = 0.61979

Reduced regular vine-copula model

First, the regular vine of the reduced model and the scatter plot and densities of the joint distribution of the reduced model are shown in Fig. 3.

Here, the decomposition in dependence trees is given in Fig. 4.

Finally, the quantification of each dependence tree with bivariate copulas is presented.

Tree: 1
5,3 <-> Gaussian, parameters = 0.377419
4,2 <-> Gaussian, parameters = 0.8069
3,2 <-> Frank, parameters = -2.41646
2,1 <-> BB7 180°, parameters = 5.70257 1e-06
Tree: 2
5,2 | 3 <-> Gaussian, parameters = 0.828941
4,3 | 2 <-> Gaussian, parameters = -0.265501
3,1 | 2 <-> Independence
Tree: 3
5,1 | 2,3 <-> BB1 180°, parameters = [1e-06, 1.23315]
4,1 | 3,2 <-> BB1 180°, parameters = [1e-06, 1.20484]
Tree: 4
5,4 | 1,2,3 <-> Clayton 180°, parameters = 0.61979

Hypothesis tests for marginal distributions

The null hypothesis of this test is that both samples come from the same distribution. Therefore, $p-values$ below the significance level (here, 0.05) indicate a statistically significant difference.

	UI-1 inbound	UI-1 outbound	UI-2	MI	LI
UI-1 inbound	-	0.99	0.08	0.06	**<0.01**
UI-1 outbound		-	0.11	0.09	**<0.01**
UI-2			-	0.74	0.08
MI				-	0.50

Table 1. P-values obtained from the Kolmogorov-Smirnov test between the different datasets for the variable ship length (L_s).

	UI-1 inbound	UI-1 outbound	UI-2	MI	LI
UI-1 inbound	-	0.98	0.23	0.32	0.26
UI-1 outbound		-	0.17	0.40	0.19
UI-2			-	0.58	0.87
MI				-	0.88

Table 2. P-values obtained from the Kolmogorov-Smirnov test between the different datasets for the variable ship width (W_s).

	UI-1 inbound	UI-1 outbound	UI-2	MI	LI
UI-1 inbound	-	0.90	0.28	0.59	0.26
UI-1 outbound		-	0.24	0.52	0.17
UI-2			-	0.17	0.62
MI				-	0.07

Table 3. P-values obtained from the Kolmogorov-Smirnov test between the different datasets for the variable ship relative velocity (V).

	UI-1 inbound	UI-1 outbound	UI-2	MI	LI
UI-1 inbound	-	0.95	0.06	0.15	0.07
UI-1 outbound		-	**0.04**	0.22	0.13
UI-2			-	0.23	0.41
MI				-	0.68

Table 4. P-values obtained from the Kolmogorov-Smirnov test between the different datasets for the variable blockage factor (C_H).

	UI-1 inbound	UI-1 outbound	UI-2	MI	LI
UI-1 inbound	-	0.13	**<0.01**	**<0.01**	**<0.01**
UI-1 outbound		-	**<0.01**	**<0.01**	**<0.01**
UI-2			-	0.77	0.34
MI				-	0.14

Table 5. P-values obtained from the Kolmogorov-Smirnov test between the different datasets for the variable primary wave height (H_p).

	UI-1 inbound	UI-1 outbound	LI
UI-1 inbound	-	0.55	**<0.01**
UI-1 outbound		-	**<0.01**

Table 6. P-values obtained from the Kolmogorov-Smirnov test between the different datasets for the variable peak energy flux (E_p).

Fit of univariate marginal distributions

Variable	Database				
	UI-1 inbound	UI-1 outbound	UI-2	MI	LI
L_s	GEV with parameters $\mu_{GEV} = 245.11$, $\sigma_{GEV} = 76.24$ and $\xi_{GEV} = -0.58$. Type III, Reverse Weibull.				
W_s	GEV with parameters $\mu_{GEV} = 33.33$, $\sigma_{GEV} = 7.86$ and $\xi_{GEV} = -0.37$. Type III, Reverse Weibull.				
V	GEV with parameters $\mu_{GEV} = 5.92$, $\sigma_{GEV} = 0.79$ and $\xi_{GEV} = -0.34$. Type III, Reverse Weibull.				
C_H	Beta, scaled with *loc*=0.01 and *scale*=0.09, and with shape parameters α=1.92, and β=1.98				
H_p	GEV with parameters $\mu_{GEV} = 47.86$, $\sigma_{GEV} = 31.34$ and $\xi_{GEV} = -0.03$. Type III, Reverse Weibull.		GEV with parameters $\mu_{GEV} = 18.15$, $\sigma_{GEV} = 11.66$ and $\xi_{GEV} = 0.34$. Type II, Frechet.		
E_p	GPD with parameters $\mu_{GPD} = 0.08$, $\sigma_{GPD} = 10.77$ and $\xi_{GPD} = -0.066$.		-	-	GPD with parameters $\mu_{GPD} = 0.36$, $\sigma_{GPD} = 2.21$ and $\xi_{GPD} = 0.41$.

Table 7. Summary of the parametric marginal distribution functions fitted to the observations.

Fig. 5 shows the fit of the Generalized Extreme Value (GEV) distribution (cumulative distribution function in Eq. (1)) with parameters given in Table 7 to the observations of L_s.

$$F(x) = exp\left(-[1+\xi_{GEV}\frac{x-\mu_{GEV}}{\sigma_{GEV}}]^{-1/\xi_{GEV}}\right) \qquad (1+\xi_{GEV}\frac{x-\mu_{GEV}}{\sigma_{GEV}}) > 0 \tag{1}$$

where μ_{GEV}, σ_{GEV} and ξ_{GEV} are the location, scale and shape parameters of the GEV distribution.

Fig. 6 presents the fit of the Generalized Extreme Value (GEV) distribution (cumulative distribution function in Eq. (1)) with parameters given in Table 7 to the observations of W_s.

Fig. 7 illustrates the fit of the Generalized Extreme Value (GEV) distribution (cumulative distribution function in Eq. (1)) with parameters given in Table 7 to the observations of V.

Fig. 8 shows the fit of the scaled Beta distribution (probability density function in Eq. (2)) with parameters given in Table 7 to the observations of C_H.

$$f(x) = \frac{\Gamma(\alpha_{beta}+\beta_{beta})x^{\alpha_{beta}-1}(1-x)^{\beta_{beta}-1}}{\Gamma(\alpha_{beta})\Gamma(\beta_{beta})} \tag{2}$$

where α_{beta} and β_{beta} are the parameters of the distribution. Note that here the scaled version of the distribution in Scipy[?] is used, so the lower boundary of the distribution is set to *loc* and the distribution spans over a range of values equal to *scale*.

Fig. 9 presents the fit of the Generalized Pareto (GPD) distribution (cumulative distribution function in Eq. (3)) with parameters given in Table 7 to the observations of E_p.

$$F(x) = \begin{cases} 1-\left(1+\frac{\xi_{GPD}(x-\mu_{GPD})}{\sigma_{GPD}}\right)^{-1/\xi_{GPD}} & for\ \xi_{GPD} \neq 0 \\ 1-exp\left(-\frac{(x-\mu_{GPD})}{\sigma_{GPD}}\right) & for\ \xi_{GPD} = 0 \end{cases} \tag{3}$$

where μ_{GPD}, σ_{GPD} and ξ_{GPD} are the location, scale and shape parameters of the GPD distribution.

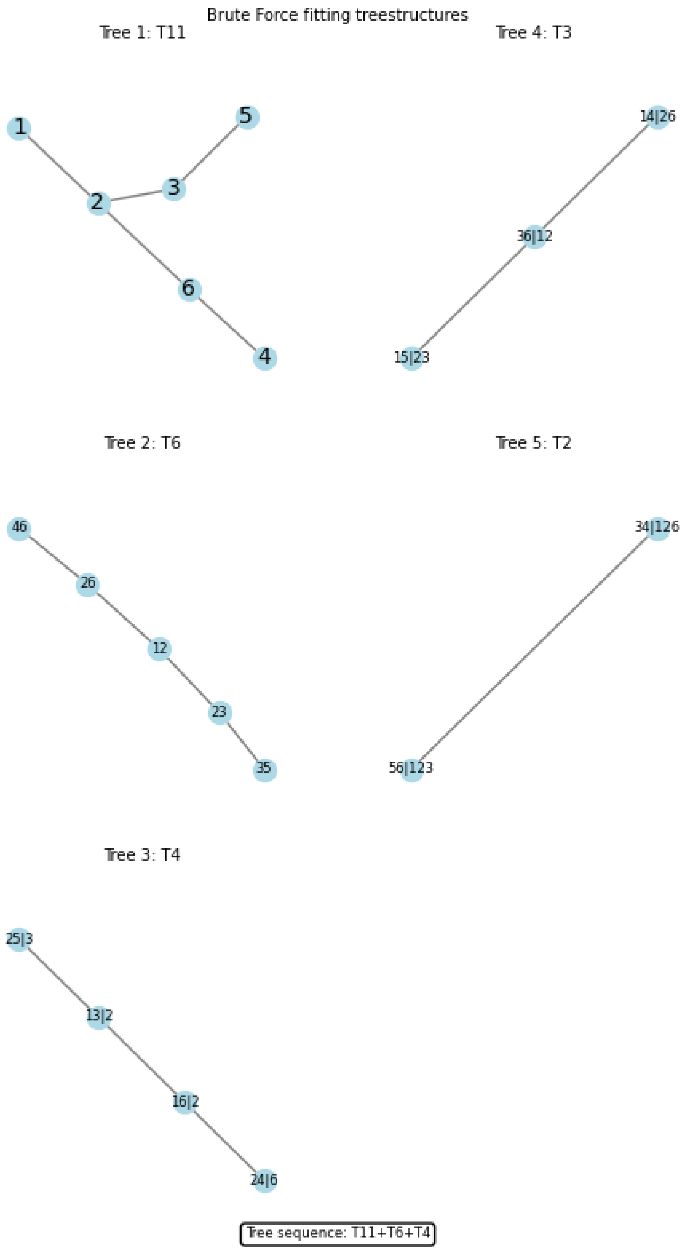

Figure 2. Decomposition of the complete regular vine-copula model in dependence trees.

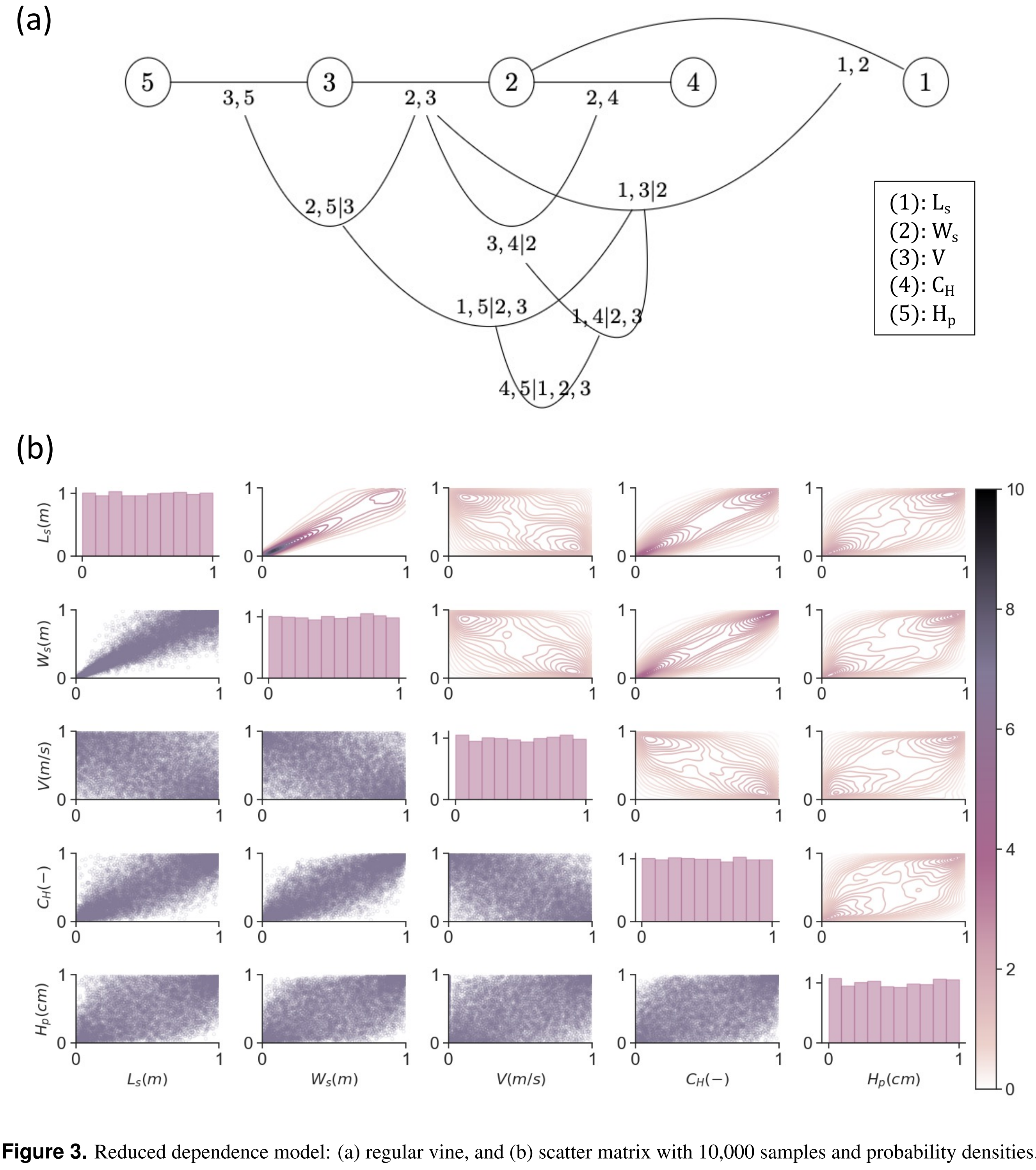

Figure 3. Reduced dependence model: (a) regular vine, and (b) scatter matrix with 10,000 samples and probability densities.

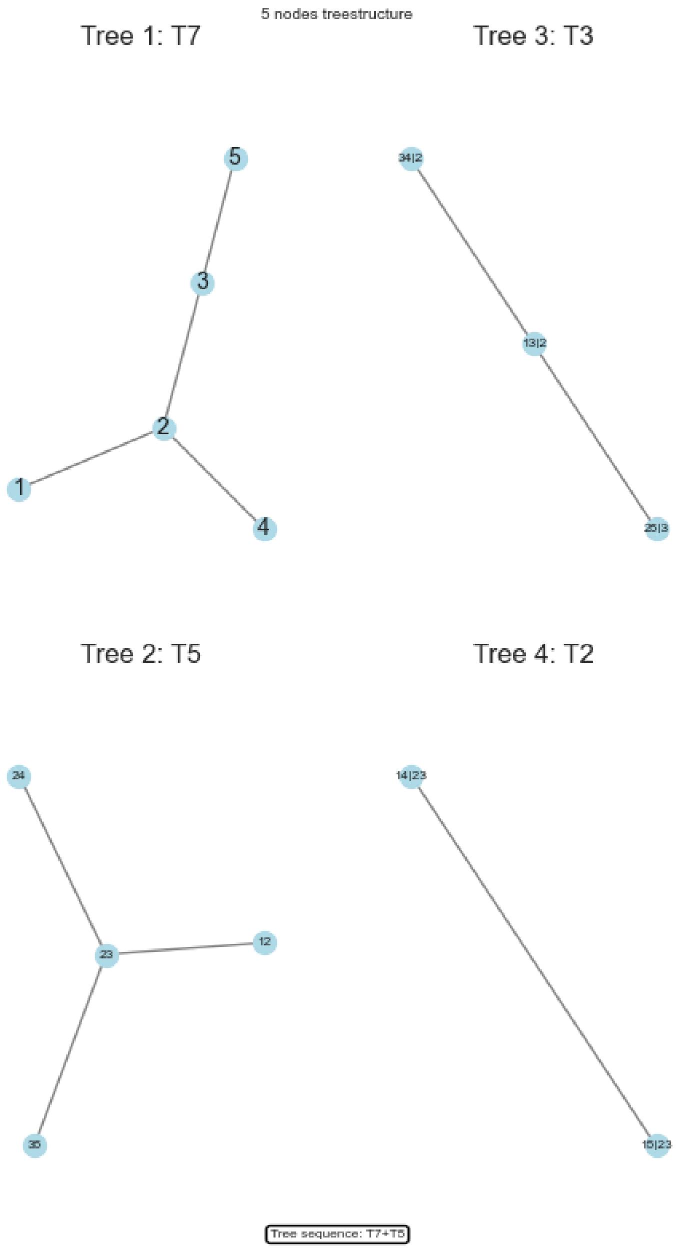

Figure 4. Decomposition of the complete regular vine-copula model in dependence trees.

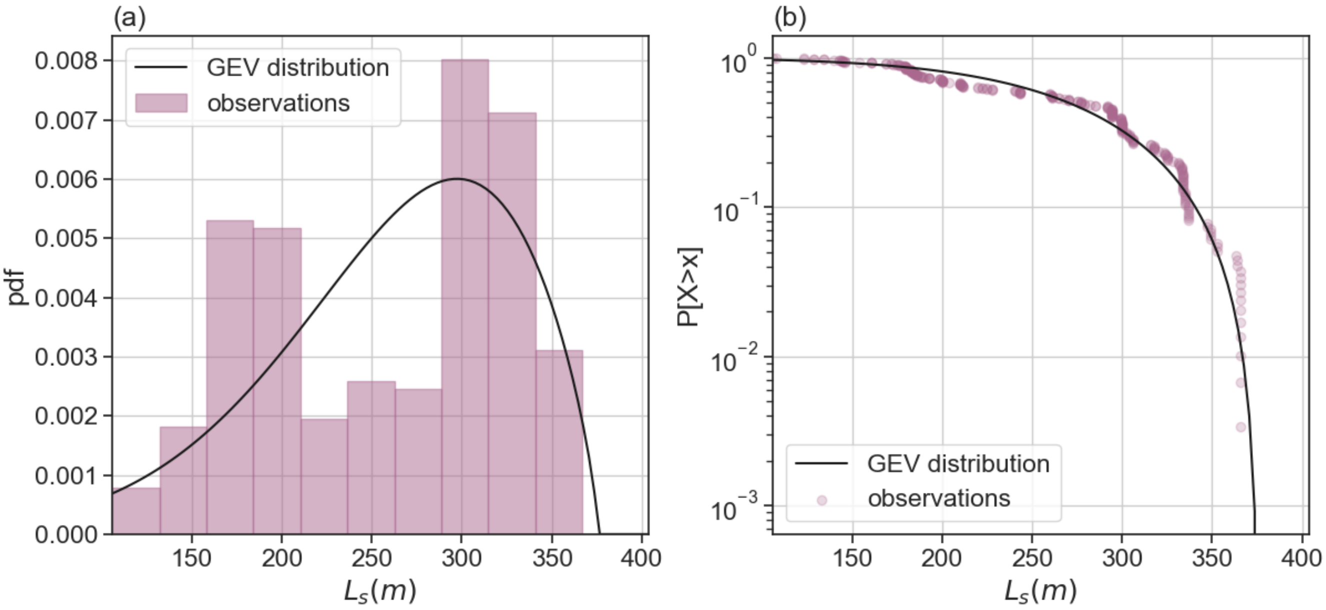

Figure 5. Comparison of the empirical and GEV parametric distribution of L_s (m/s): (a) probability density function, and (b) exceedance plot in semi-log scale.

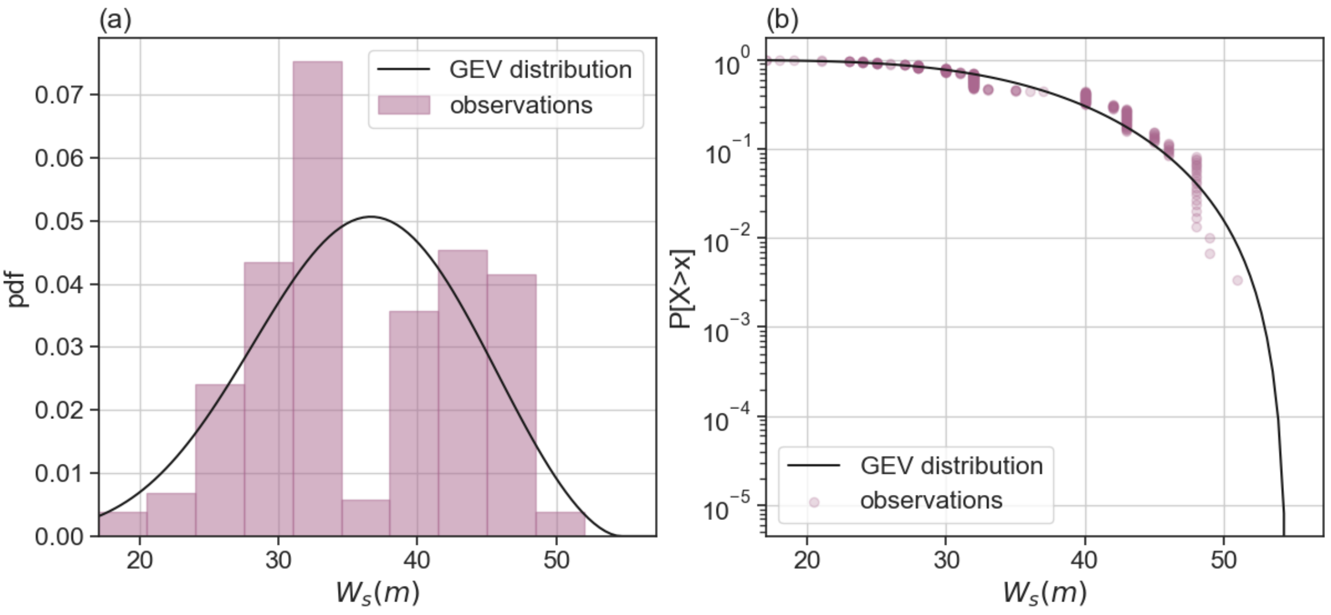

Figure 6. Comparison of the empirical and GEV parametric distribution of W_s (m/s): (a) probability density function, and (b) exceedance plot in semi-log scale.

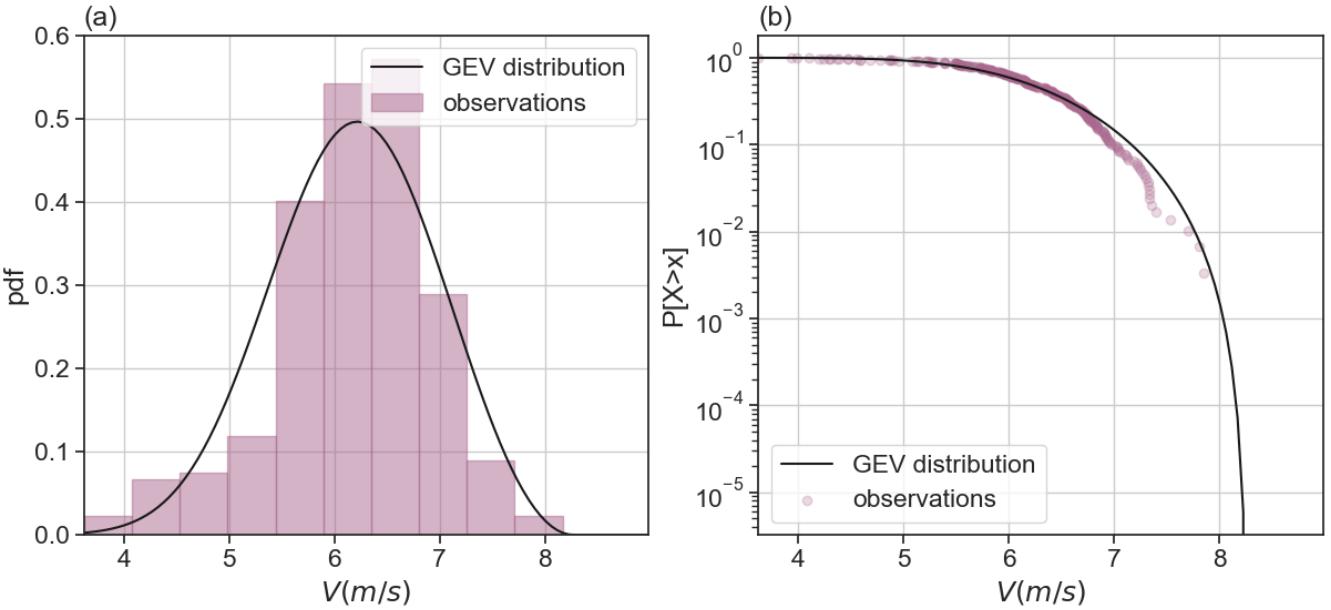

Figure 7. Comparison of the empirical and GEV parametric distribution of V (m/s): (a) probability density function, and (b) exceedance plot in semi-log scale.

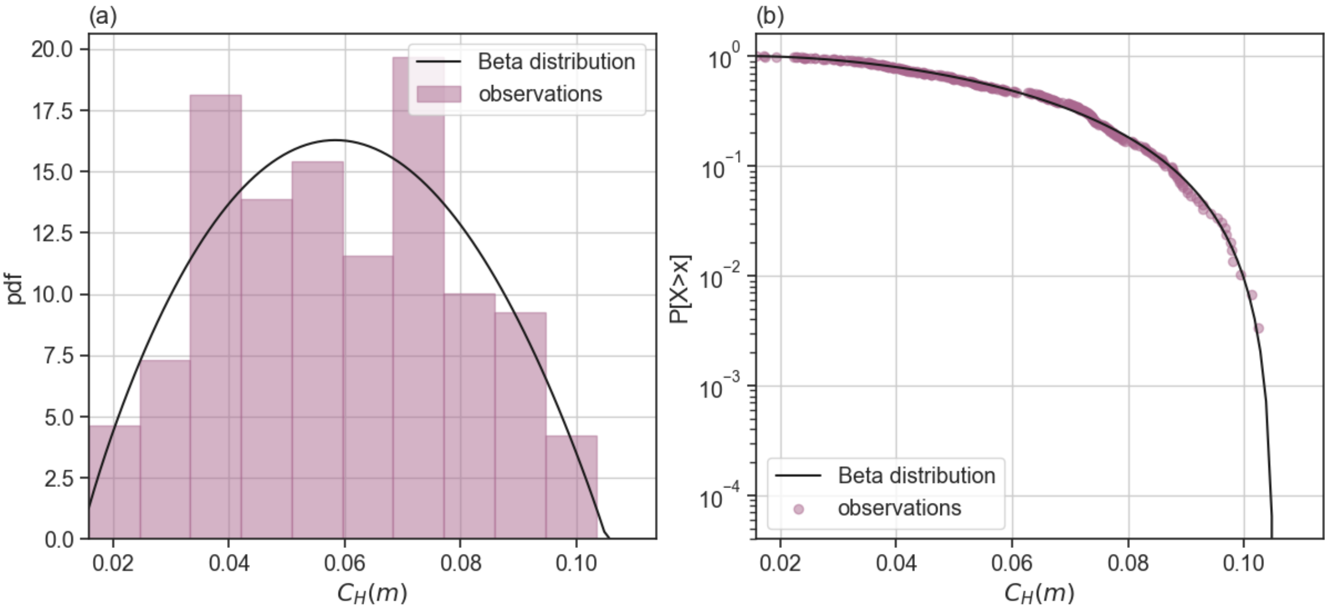

Figure 8. Comparison of the empirical and Beta parametric distribution of C_H (m/s): (a) probability density function, and (b) exceedance plot in semi-log scale.

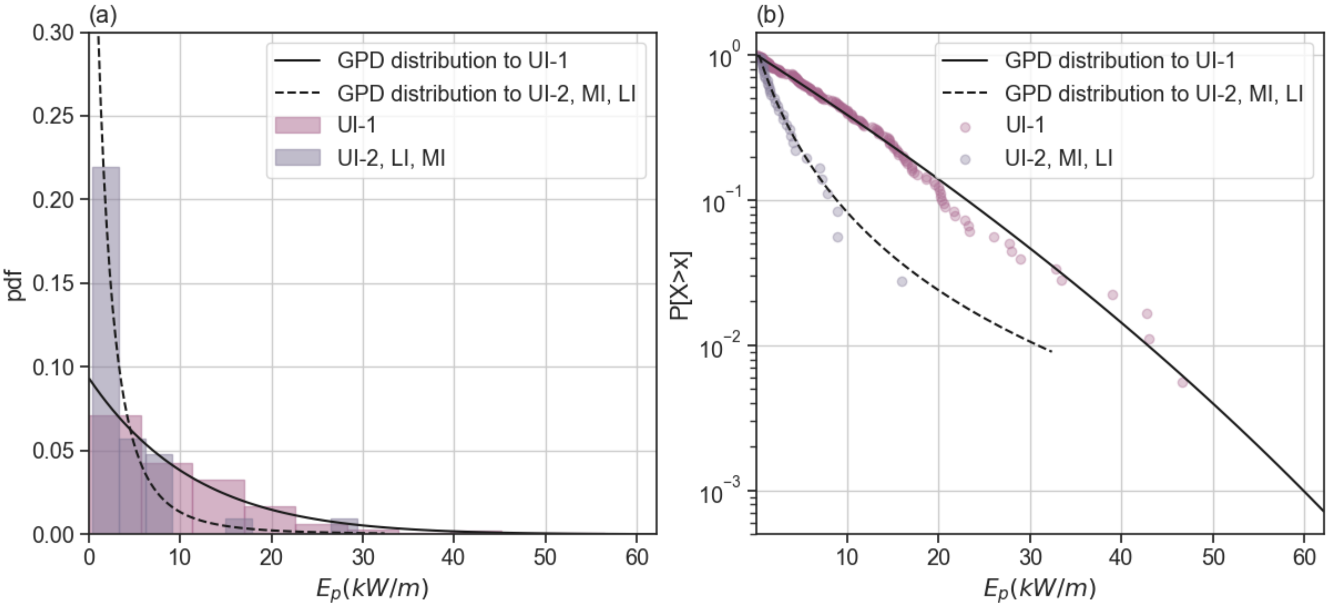

Figure 9. Comparison of the empirical and GPD parametric distribution of E_p (cm): (a) probability density function, and (b) exceedance plot in semi-log scale.
